# Supplementary material for: Molecular basis of β-lactam antibiotic resistance of ESKAPE bacterium E. faecium Penicillin Binding Protein PBP5
Source: Nat Commun. 2023 Jul 17;14:4268. doi: 10.1038/s41467-023-39966-5 (PMC10352307; doi:10.1038/s41467-023-39966-5)
Supplement: Supplementary file 1 — Supplementary Information [file 41467_2023_39966_MOESM1_ESM.pdf]

Supplemental Material for

Molecular basis of β-lactam Antibiotic resistance of ESKAPE bacterium *E. faecium* Penicillin Binding Protein PBP5

Yamanappa Hunashal[1], Ganesan Senthil Kumar[1,2], Meng S. Choy[1], Éverton D. D'Andréa[3], Andre Da Silva Santiago[4], Marta V. Schoenle[3], Charlene Desbonnet[5], Michel Arthur[6], Louis B. Rice[5], Rebecca Page[4] & Wolfgang Peti[1,*]

[1]Department of Molecular Biology and Biophysics, University of Connecticut Health Center, Farmington, USA; [2]National Institute of Immunology, New Delhi, India; [3]Department of Chemistry and Biochemistry, University of Arizona, Tucson, USA; [4]Department of Cell Biology, University of Connecticut Health Center, Farmington, USA; [5]Department of Medicine, Rhode Island Hospital, Warren Alpert Medical School of Brown University, Providence, USA; [6]INSERM, Sorbonne Université, Université Paris Cité, Paris, France.

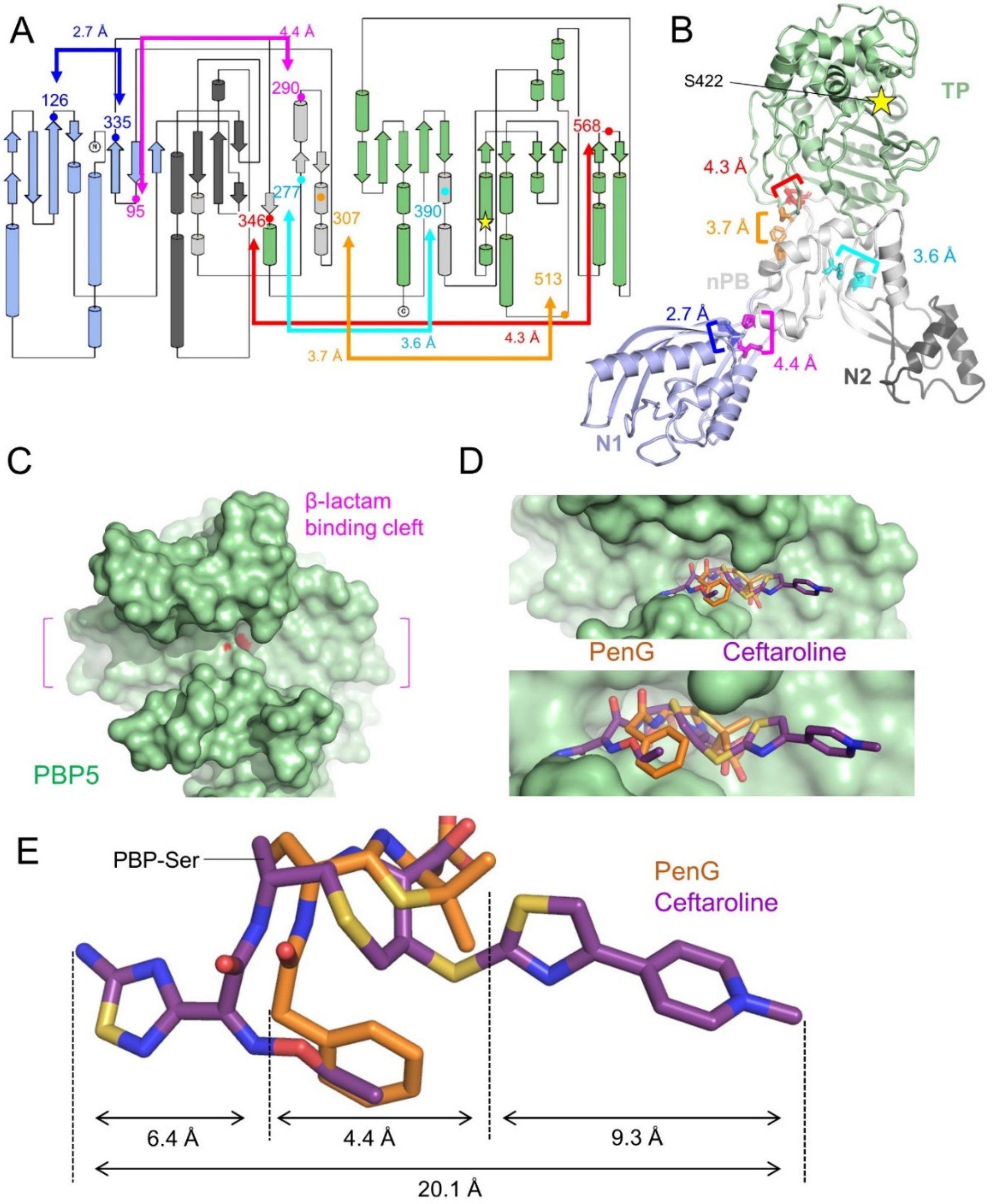

Supplementary Figure 1. PBP5 β-lactam binding cleft. (**A**) PBP5 topology map showing distinct secondary structural elements (arrows, β-strands, cylinders, α-helices) and colored according to domain (N1, light blue; N2, dark grey; nBP, light grey; TP, light green), with residues close in space but distant in sequence indicated by colored arrows, and the measured Cα-Cα distances indicated. (**B**) PBP5 shown as a cartoon with domains colored as in (A). The location of the residues highlighted in (A) along with measured distances shown. (**C**) PBP5 (green surface), with catalytic S422 shown in red. The location of the deep cleft into which β-lactams bind is indicated by a pink bracket. (**D**) Overlay of PBP5-penG (PBP5, green surface, penG, orange sticks, PDBid 6BSR) and PBP4-ceftaroline (superimposed using the TP domains; ceftaroline, violet sticks, PDBid 6MKI). (**E**) Overlay Identical to that shown in (B). PenG forms a compact "U" shape with a ~5 Å overall width. In contrast, ceftaroline is much longer (~20 Å) as it is extended on one side via a thiazole and a pyridine ring (~9.3 Å) and an oxime group and thiazole ring moieties (~6.4 Å) on other side of the core β-lactam ring.

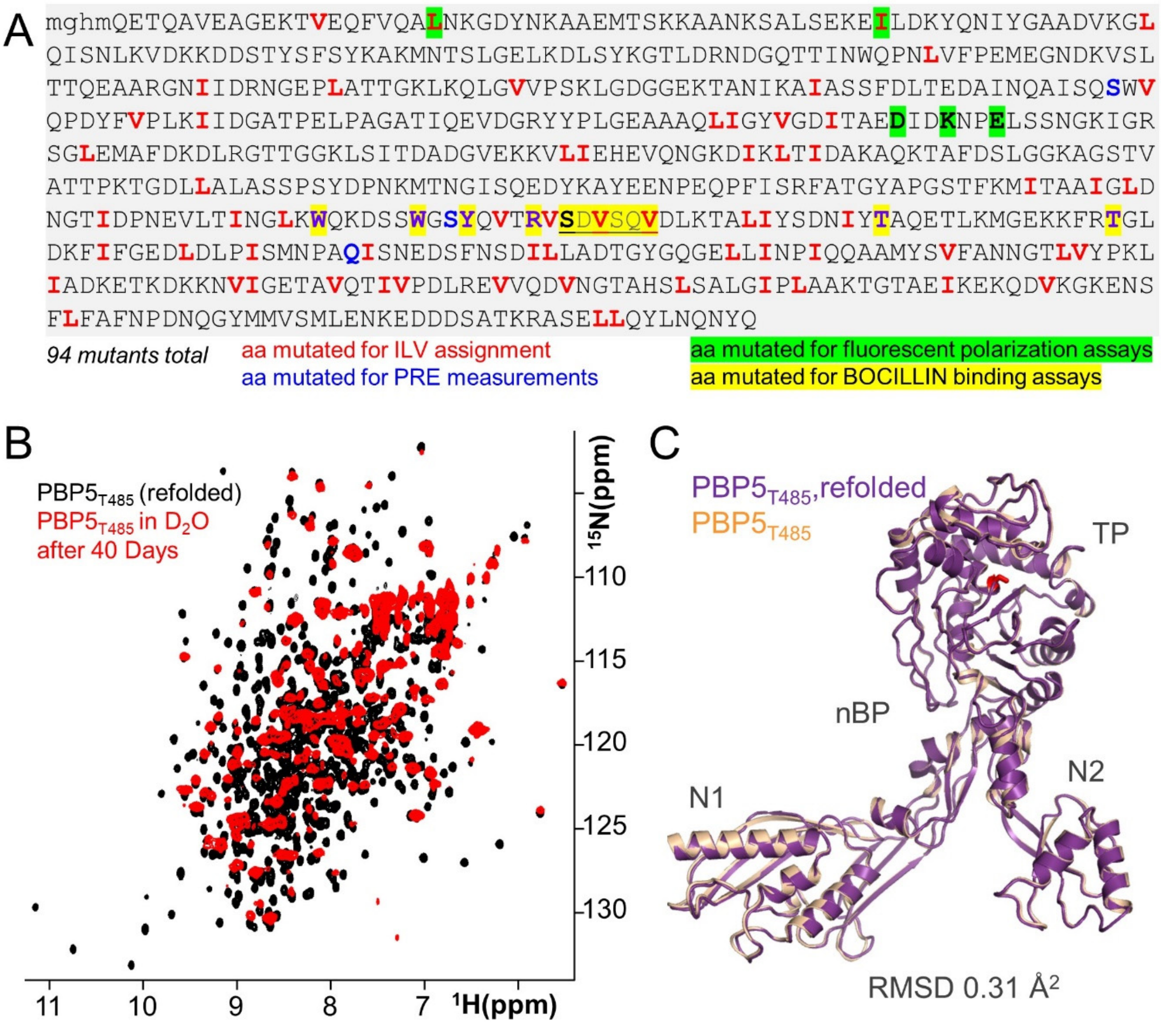

Supplementary Figure 2: PBP5 data. (**A**) $PBP5_{T485}$ sequence. Different variants used for assignment, structural, biochemical and functional studies are highlighted. (**B**) Hydrogen/deuterium exchange of $PBP5_{T485}$; 2D [^1H,^{15}N] TROSY spectrum of $PBP5_{T485}$ (black) and lyophilized PBP5 undergoing H/D exchange after 40 days resuspended in 100% D_2O (red). (**C**) Overlay of the crystal structure of the denatured/refolded $PBP5_{T485}$ and the natively purified $PBP5_{T485}$ structure.

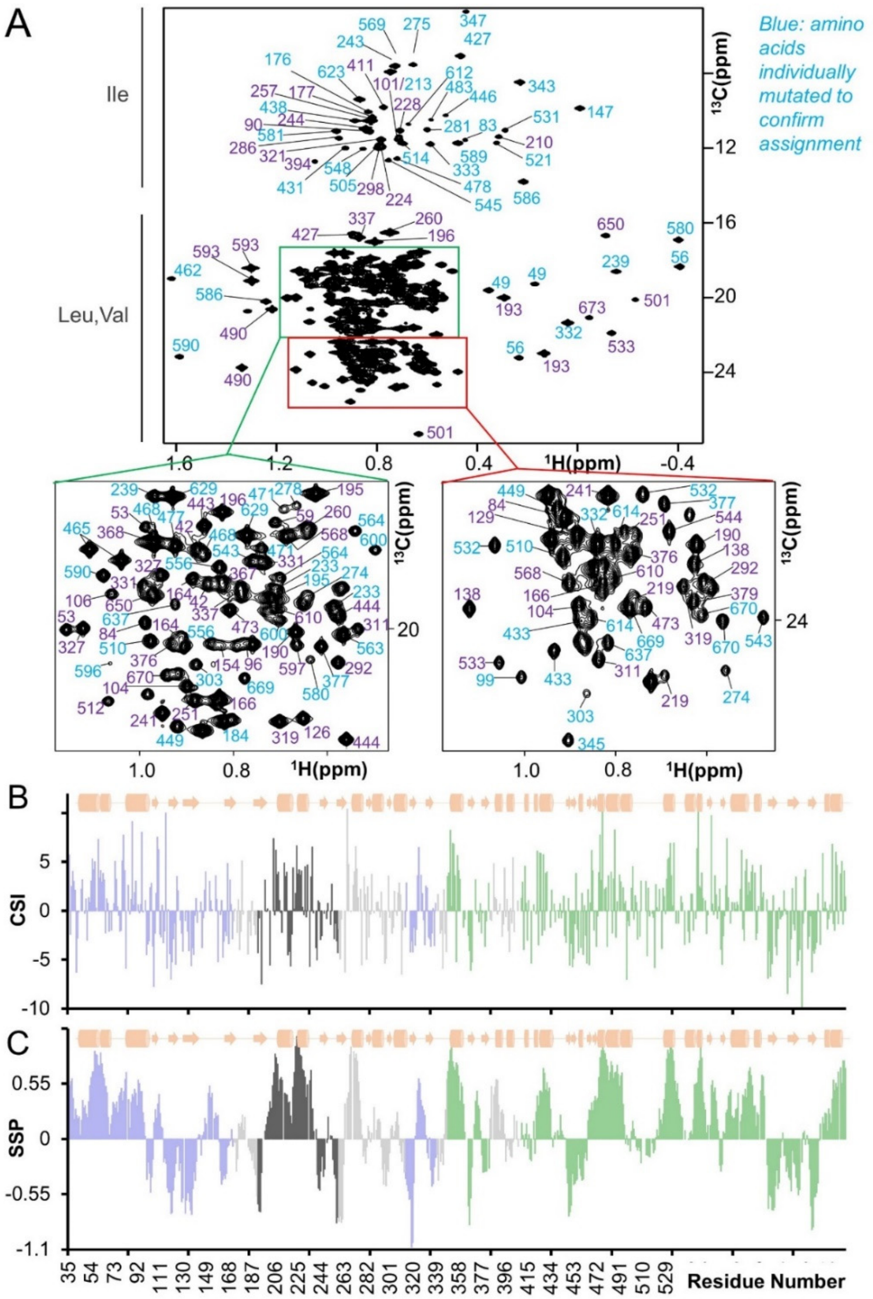

Supplementary Figure 3: PBP5$_{T485}$ ILV assignment and Chemical Shift Index. (**A**) Fully-annotated 2D [^{1}H,^{13}C] HMQC spectrum of ILV PBP5$_{T485}$. Chemical shift sections for Ile, Val and Leu are highlighted by black bars (left); green and red insert highlight crowded Val/Leu regions. Residues in blue letters are confirmed by single point variants of PBP5. (**B**) Chemical shift Index (CSI) for PBP5$_{T485}$ (blue: N1 domain; black: N2 domain; gray: nPB domain; green; TP domain). CSI compares the experimental Cα and Cβ chemical shifts of PBP5$_{T485}$ to random coil chemical shifts (RefDB database); positive or negative deviations correlate with α-helical or β-sheet secondary structure. Secondary structure elements from PBP5 crystal structure (PDBid: 6MKA) are plotted above. (**C**) Secondary-structure propensity (SSP) data for PBP5 plotted vs. residue numbers. (SSP > 0, α helix; SSP < 0, β strand). Cα and Cβ chemical shifts were used to create the CSI and SSP plots in A/B (RefDB database). Source data are provided as a Source Data file.

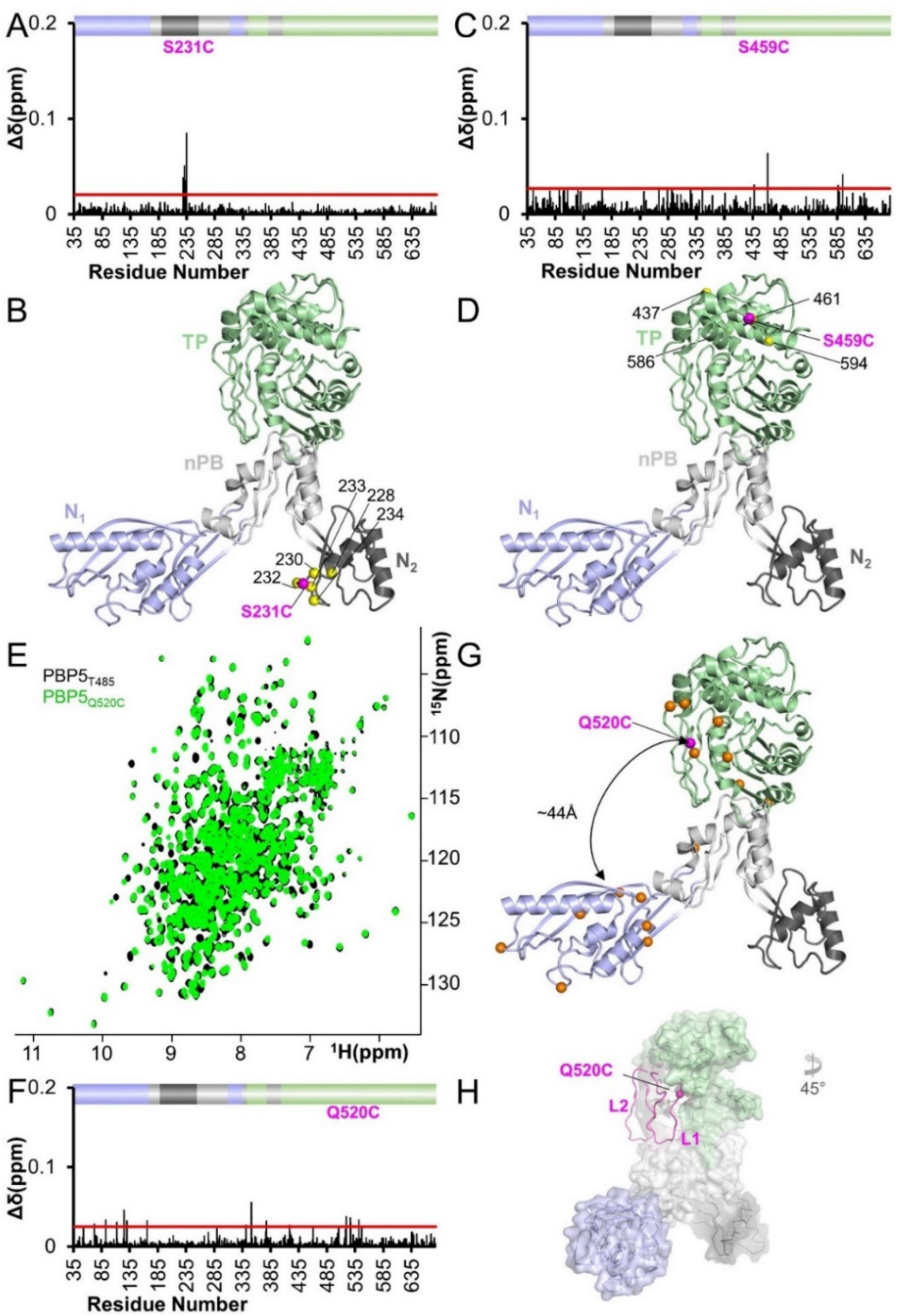

Supplementary Figure 4: PBP5 solution state topology. (**A**) Chemical shift perturbation (CSP) vs residue number plot of PBP5 S231C, bar colored according to PBP5 domain structure. (**B**) PBP5 S231C CSP plotted on 3D PBP5 structure (PDBid: 6MKA); residues with ≥avg ± 3*std (red line in A/B) highlighted. (**C**) CSP vs residue number plot of PBP5 S459C, bar colored according to PBP5 domain structure. (**D**) PBP5 S459C CSP plotted on 3D PBP5 structure; identical as for C. (**E**) Overlay of 2D [^{1}H,^{15}N] TROSY spectrum of PBP5 (black) and PBP5 Q520C (green). (**F**) CSP vs residue number plot of PBP5 Q520C, bar colored according to PBP5 domain structure. (**G**) PBP5 Q520C CSP plotted on 3D PBP5 structure; identical as for F. (**H**) Surface representation of PBP5 Q520C; Q520C highlighted in magenta; Loops L1 and L2 highlighted. Source data are provided as a Source Data file.

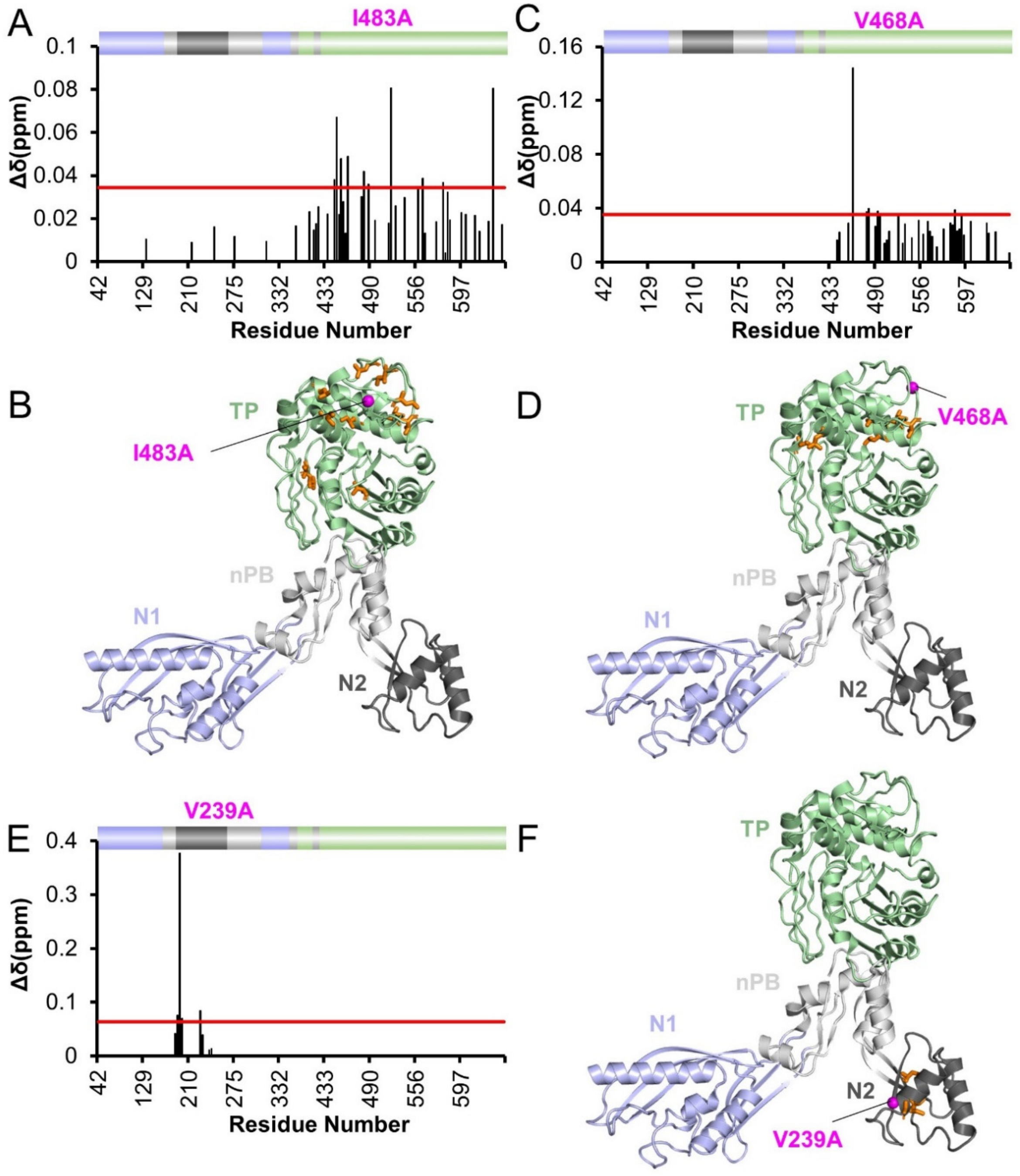

Supplementary Figure 5: **The TP and the N2 domains of PBP5 are independent of nPB and N1**. (**A**) Chemical shift perturbation (CSP) vs residue number plot of PBP5 I483A, bar colored according to PBP5 domain structure. (**B**) PBP5 I483A CSP plotted on 3D PBP5 structure (PDBid: 6MKA); residues with ≥avg ± 3*std (red line in A) highlighted. (**C**) CSP vs residue number plot of PBP5 V468A, bar colored according to PBP5 domain structure. (**D**) PBP5 V468A CSP plotted on 3D PBP5 structure (PDBid: 6MKA); residues with ≥avg ± 3*std (red line in C) highlighted. (**E**) CSP vs residue number plot of PBP5 V239A, bar colored according to PBP5 domain structure. (**F**) PBP5 V239A CSP plotted on 3D PBP5 structure (PDBid: 6MKA); residues with ≥avg ± 3*std (red line in E) highlighted. Source data are provided as a Source Data file.

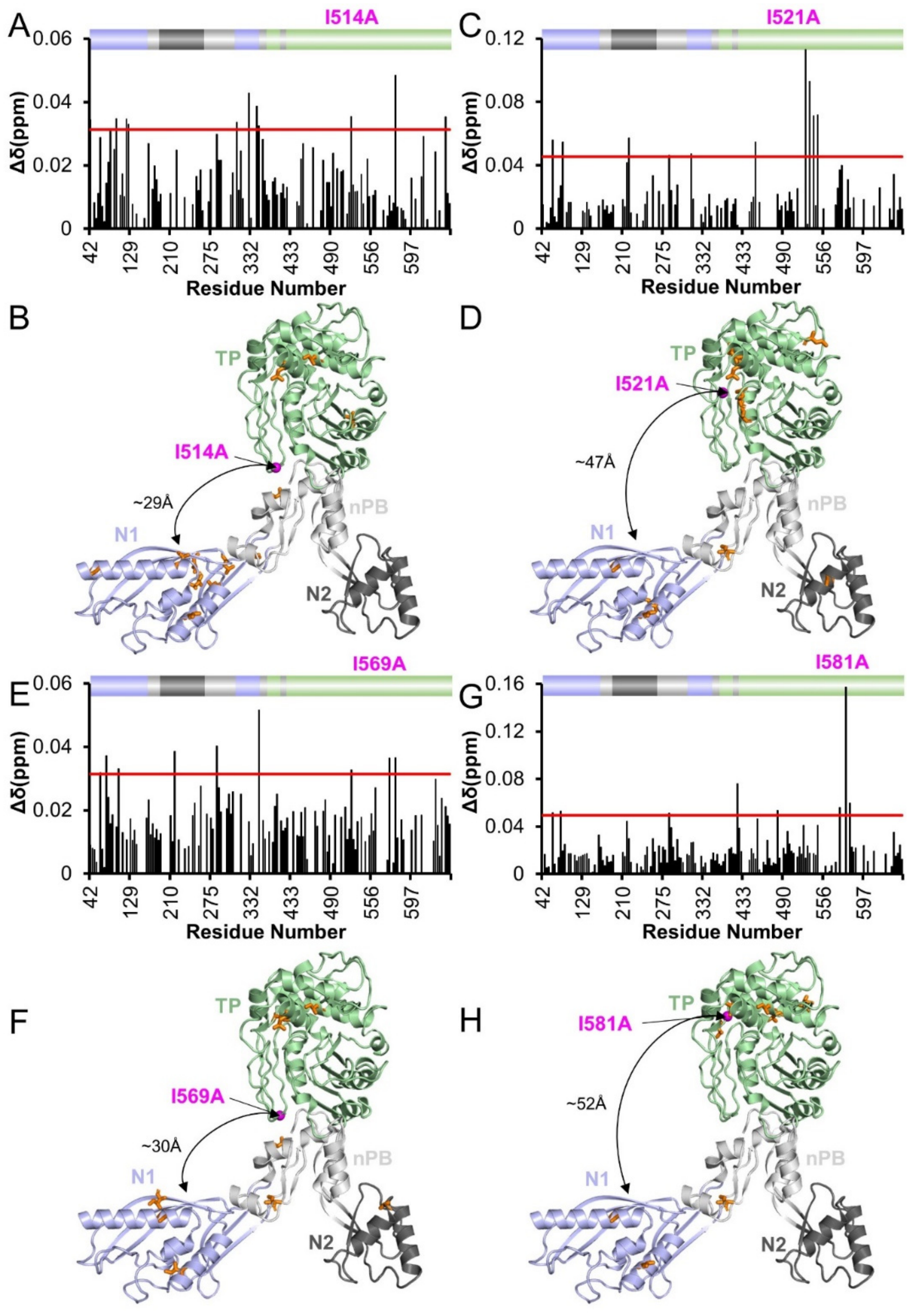

Supplementary Figure 6: PBP5 TP and N1 domain are connected. (**A**) Chemical shift perturbation (CSP) vs residue number plot of PBP5 I514A, bar colored according to PBP5 domain structure. (**B**) PBP5 I514A CSP plotted on 3D PBP5 structure (PDBid: 6MKA); residues with ≥avg ± 2*std (red line in A) highlighted. (**C**) CSPs vs residue number plot of PBP5 I521A, bar colored according to PBP5 domain structure. (**D**) PBP5 I521A CSP plotted on 3D PBP5 structure (PDBid: 6MKA); residues with ≥avg ± 2*std (red line in C) highlighted. (**E**) CSPs vs residue number plot of PBP5 I569A, bar colored according to PBP5 domain structure. (**F**) PBP5 I569A CSP plotted on 3D PBP5 structure (PDBid: 6MKA); residues with ≥avg ± 2*std (red line in E) highlighted. (**G**) CSPs vs residue number plot of PBP5 I581A, bar colored according to PBP5 domain structure. (**H**) PBP5 I581A CSP plotted on 3D PBP5 structure (PDBid: 6MKA); residues with ≥avg ± 2*std (red line in G) highlighted. Source data are provided as a Source Data file.

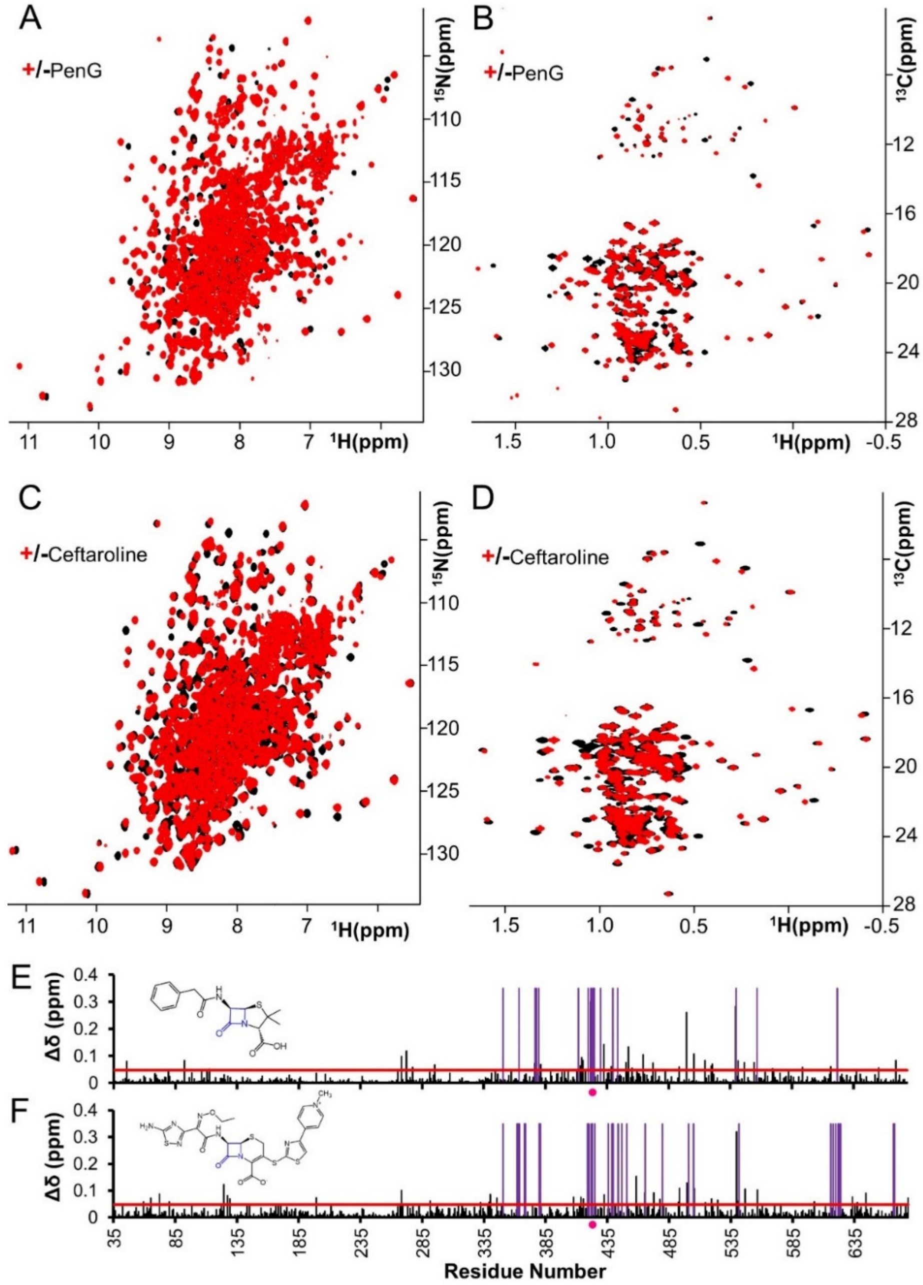

Supplementary Figure 7. **PBP5 interaction with β-lactam antibiotics.** (**A**) 2D [^{1}H,^{15}N] TROSY spectrum of PBP5 (black) and PBP5:penG (red) in saturating conditions (1:8 ratio). (**B**) 2D [^{1}H,^{13}C] ILV HMQC spectrum of PBP5 (black) and PBP5:penG (red) in saturating conditions (1:8 ratio). (**C**) 2D [^{1}H,^{15}N] TROSY spectrum of PBP5 (black) and PBP5:ceftaroline (red) in saturating conditions (1:8 ratio). (**D**) 2D [^{1}H,^{13}C] ILV HMQC spectrum of PBP5 (black) and PBP5:ceftaroline (red) in saturating conditions (1:8 ratio). (**E**) ^{15}N CSPs vs residue number for PBP5:penG (1:8 ratio); average+1σ (red line); chemical shifts changed beyond detectability (violet). Insert: penG. Red dot highlights S422. (**F**) ^{15}N CSPs vs residue number for PBP5:ceftaroline (1:8 ratio); average+1σ (red line); chemical shifts changed beyond detectability (violet). Insert: ceftaroline. Red dot highlights S422. Source data are provided as a Source Data file.

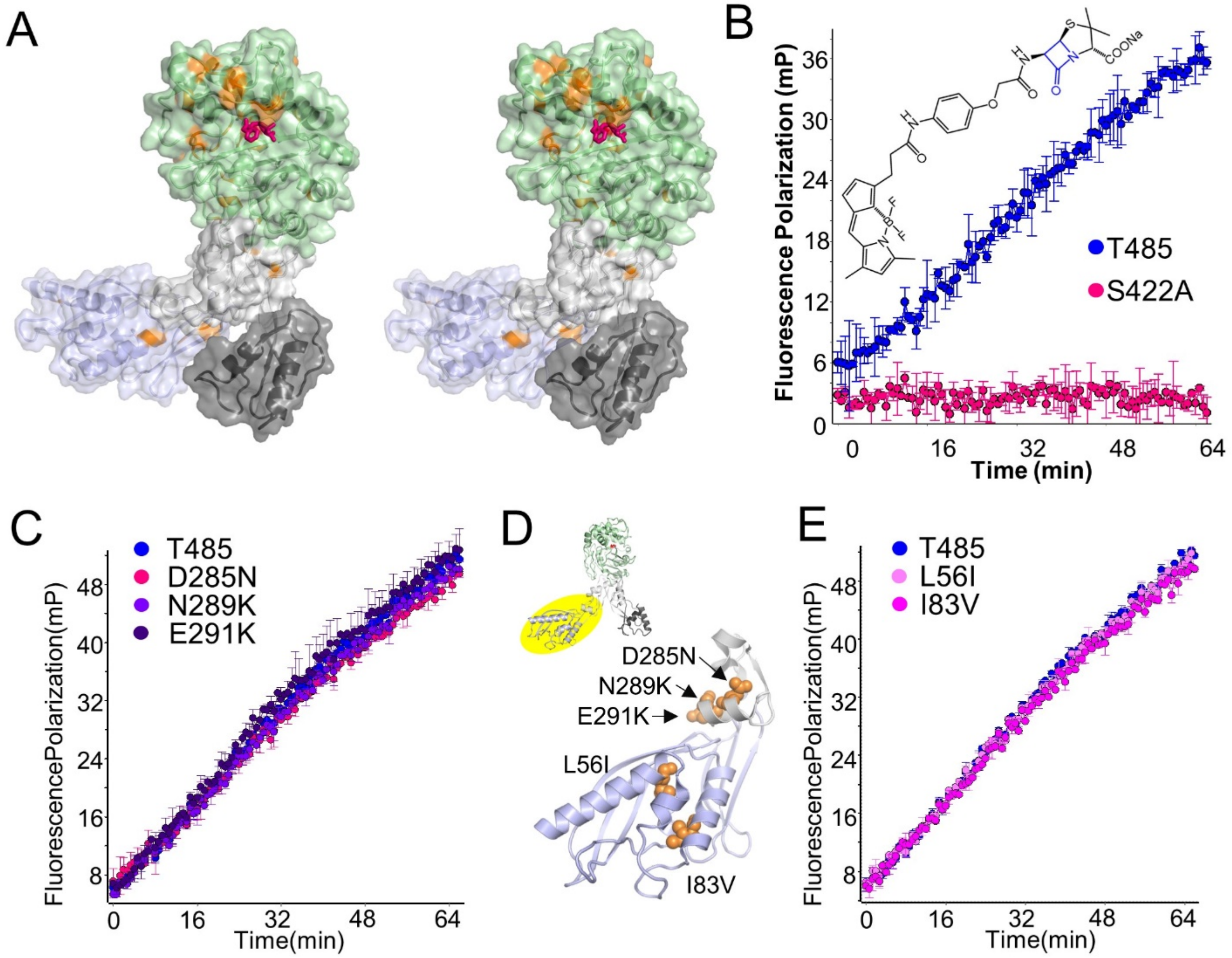

Supplementary Figure 8. **PBP5 has a single β-lactam binding pocket.** (**A**) PenG CSP mapped on PBP5 structure (PDBid: 6MKA); colors according to Fig. 1A/B; stereo view. Orange surface: residues with significant changes. (**B**) BOCILLIN FL fluorescence polarization assay with T485, blue, and S422A, pink. n=3; average ± std deviation. (**C**) BOCILLIN FL fluorescence polarization assay for PBP5 (T485: blue; D285N: red; N289K: violet; E291K, black). n=3; average ± std deviation. (**D**) PBP5 variants used in (C) and (E) are highlighted on the PBP5 structure of N1 domain; insert shows full PBP5 and N1 is highlighted in yellow. (**E**) BOCILLIN FL fluorescence polarization assay for PBP5 (T485: blue; L56I: pink; I83V: violet). n=3; average ± std deviation. Source data are provided as a Source Data file.

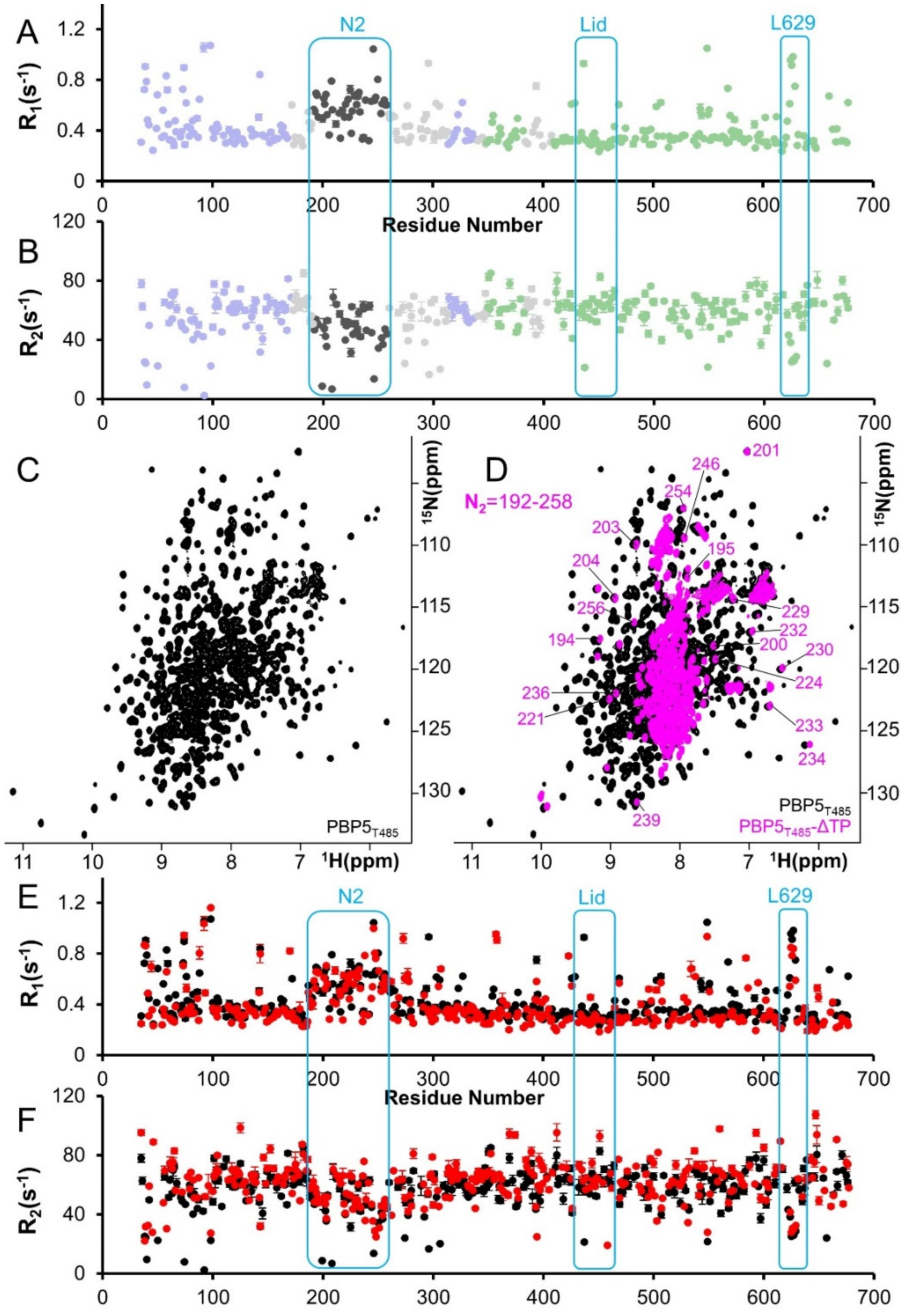

Supplementary Figure 9: ^{15}N ps/ns backbone dynamics of PBP5. (**A**) PBP5 ^{15}N R_1 vs residue number. Error bars are based on repeat measurements and are often smaller than the symbols used. average ± std deviation. (**B**) PBP5 ^{15}N R_2 vs residue number; N2, Lid and L629 are highlighted by blue boxes. Error bars are based on repeat measurements and are often smaller than the symbols used. average ± std deviation. (**C**) 2D [^{1}H,^{15}N] TROSY spectrum of PBP5$_{T485}$. (**D**) Overlay of 2D [^{1}H,^{15}N] TROSY spectrum of PBP5$_{T485}$ (black) and PBP5-ΔTP (violet). (**E**) Overlay of PBP5 ^{15}N R1 in the absence (black) and presence (red) of penG vs residue number. Error bars are based on repeat measurements and are often smaller than the symbols used. average ± std deviation. (**F**) Overlay of PBP5 ^{15}N R2 in the absence (black) and presence (red) of penG vs residue number N2, Lid and L629 are highlighted by blue boxes. Error bars are based on repeat measurements and are often smaller than the symbols used. average ± std deviation. Source data are provided as a Source Data file.

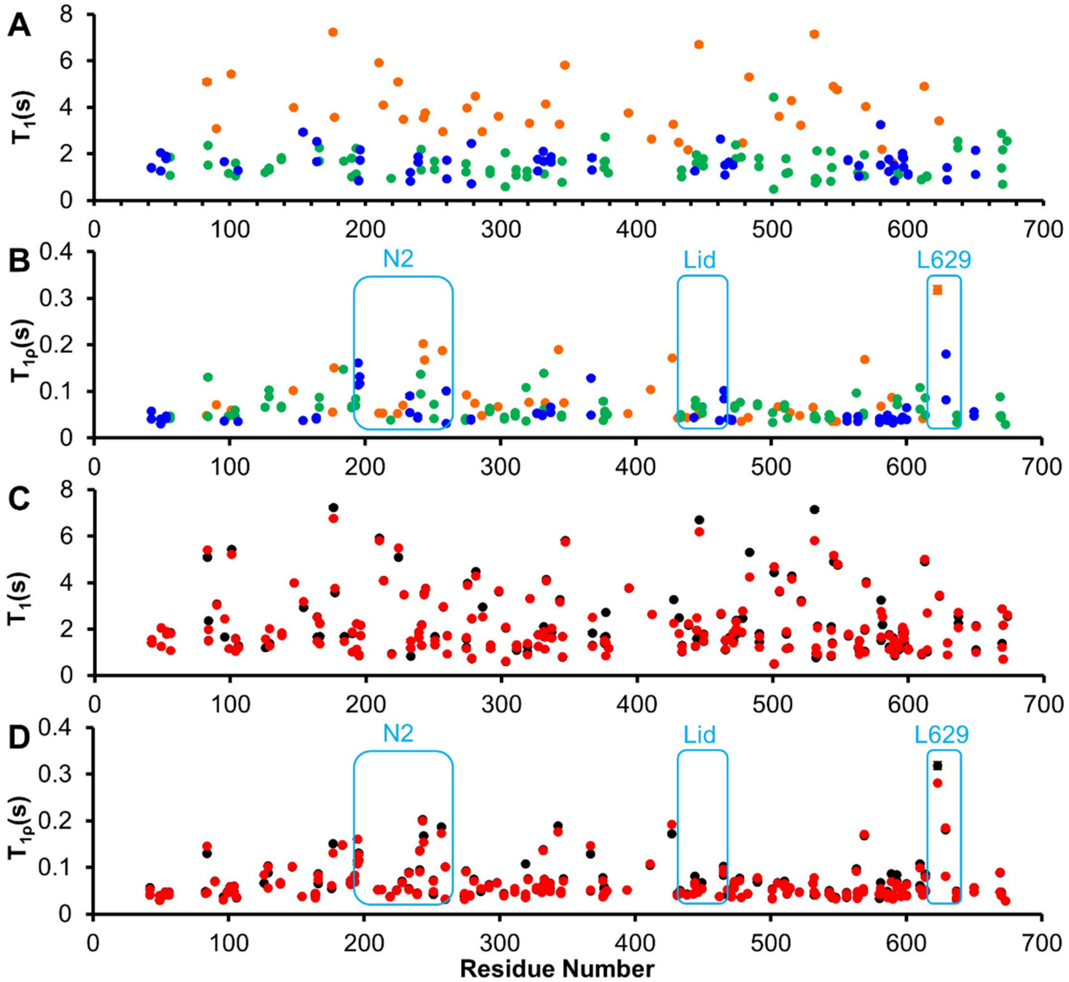

Supplementary Figure 10: ^{13}C ILV methyl dynamics of PBP5$_{T485}$. (**A**) PBP5 ^{13}C ILV T_1 vs residue number (Ile: orange; Val: blue; Leu: green). (**B**) PBP5 ^{13}C ILV $T_{1\rho}$ vs residue number, N2, Lid and Loop629 are highlighted by boxes. (**C**) Overlay of PBP5 ^{13}C ILV T_1 in the absence (black) and presence of penG (red), (**D**) Overlay of PBP5 ^{13}C ILV $T_{1\rho}$ in the absence (black) and presence of penG (red), N2, Lid and Loop629 are highlighted by boxes. Error bars are based on repeat measurements and are often smaller than the symbols used. average ± std deviation for all data shown. Source data are provided as a Source Data file.

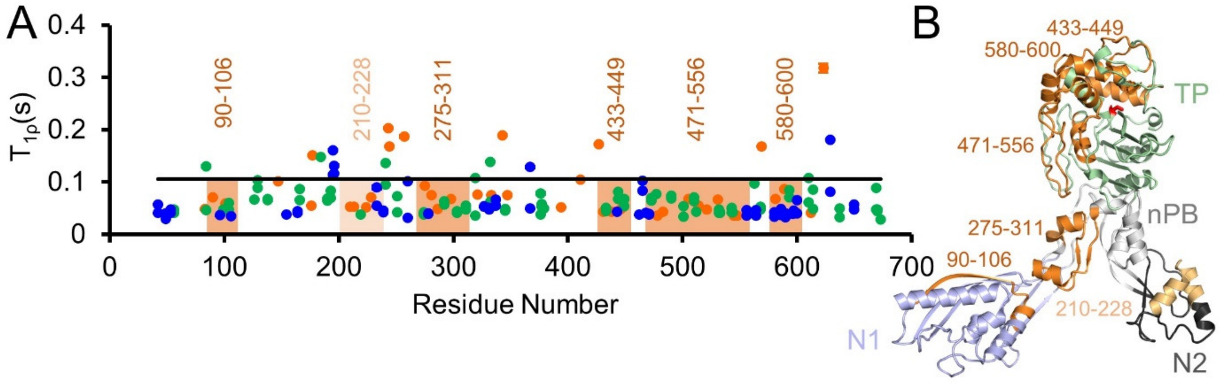

Supplementary Figure 11: Dynamic domains in PBP5. PBP5 ^{13}C ILV methyl $T_{1\rho}$ measurements highlighted differences in local mobility in different areas/domains of PBP5. Black line indicates avg+std and highlighted residues with higher-than-average rigidity: N1 domain: residues 90-106, N2 domain: residues 210-228, nPB domain: residues 275-311, TP domain: residues 433-449, 471-556, 580-600. Interestingly L1, which is part of residues 471-556, which forms a region of high rigidity is very close in space to rigid parts of the nPB domain, as shown on the 3D structure (see also Fig. S3). Error bars are based on repeat measurements and are often smaller than the symbols used. average ± std deviation.

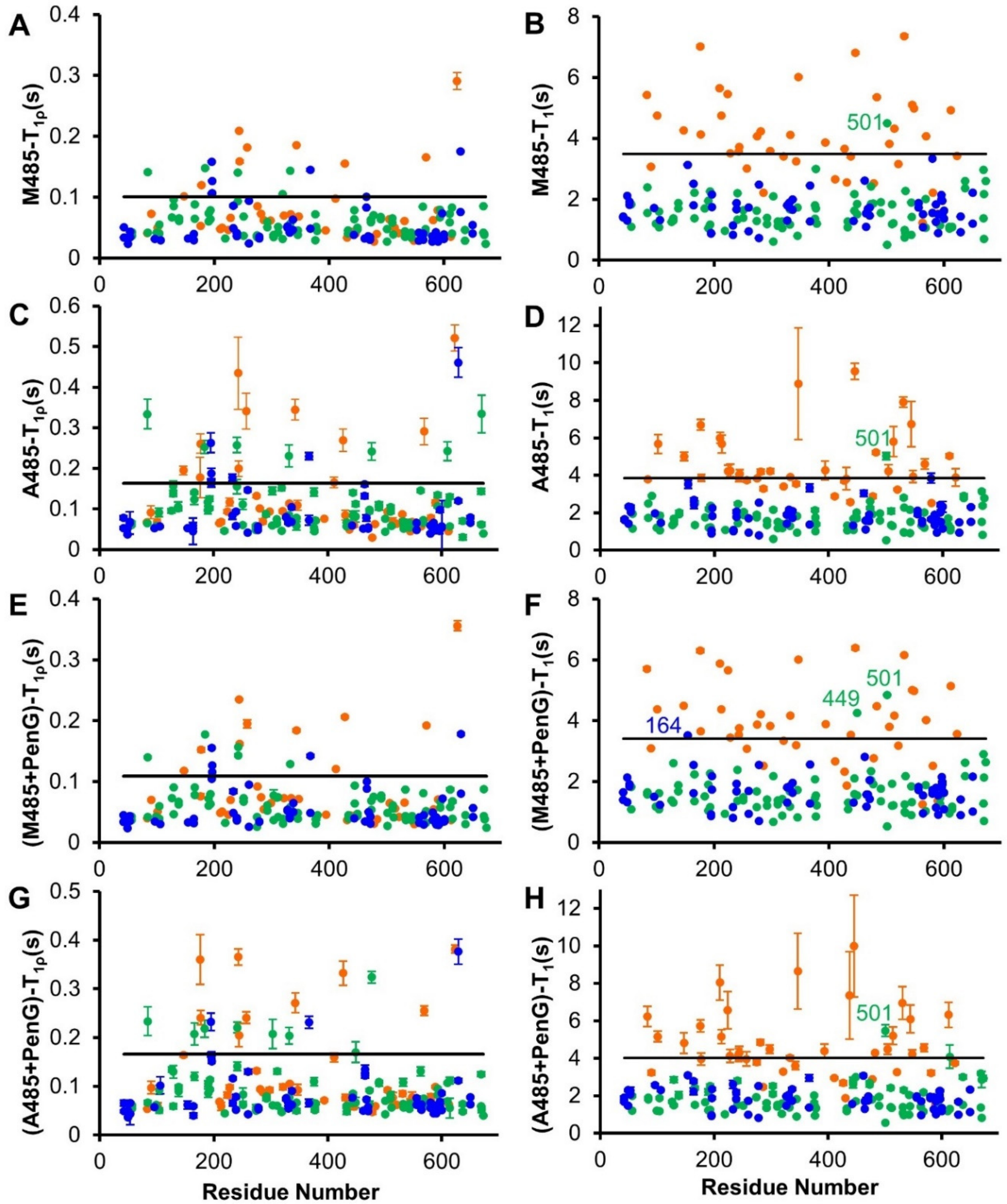

Supplementary Figure 12: ^{13}C ILV T1 and $T_{1\rho}$ of PBP5 M485 and A485 in the presence and absence of penG. (**A**) PBP5 M485 ^{13}C ILV $T_{1\rho}$ vs residue number. (**B**) PBP5 M485 ^{13}C ILV T_1 vs residue number. (**C**) PBP5 A485 ^{13}C ILV $T_{1\rho}$ vs residue number. (**D**) PBP5 A485 ^{13}C ILV T_1 vs residue number. (**E**) PBP5 M485+saturating penG ^{13}C ILV $T_{1\rho}$ vs residue number. (**F**) PBP5 M485+saturating penG ^{13}C ILV T_1 vs residue number. (**G**) PBP5 A485+saturating penG ^{13}C ILV $T_{1\rho}$ vs residue number. (**H**) PBP5 A485+saturating penG ^{13}C ILV T_1 vs residue number. All graphs: Ile: orange; Val: blue; Leu: green; black line average + standard deviation of $T_{1\rho}$. Error bars are based on repeat measurements and are often smaller than the symbols used. average ± std deviation. Source data are provided as a Source Data file.

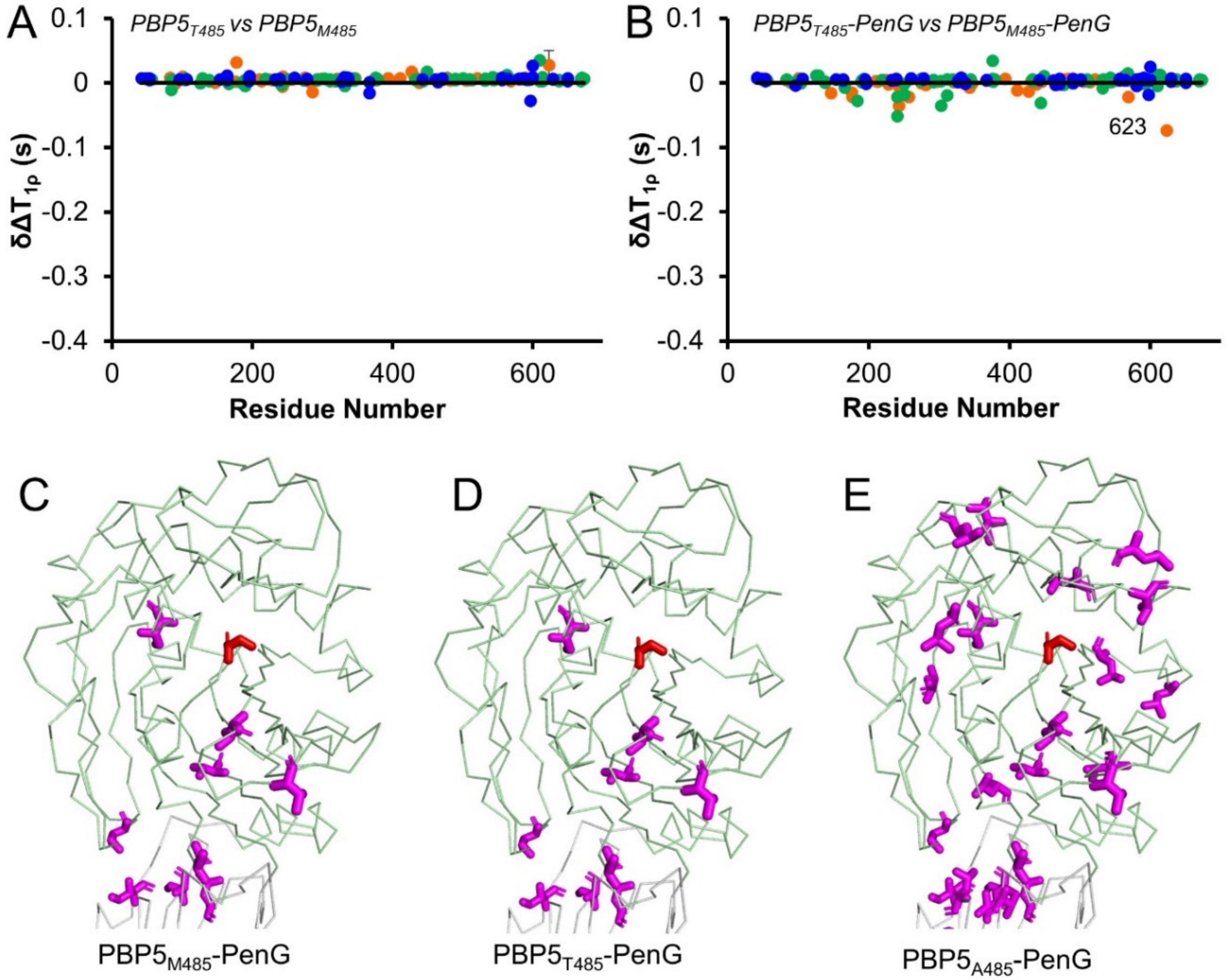

Supplementary Figure 13: Changes in PBP5 fast timescale dynamics. (**A**) ^{13}C ILV side chain dynamics ($T_{1\rho}$) comparison between $PBP5_{M485}$ and $PBP5_{T485}$ (delta $T_{1\rho}$ $PBP5_{T485}$ - $T_{1\rho}$ $PBP5_{M485}$). Error bars are based on repeat measurements and are often smaller than the symbols used. average ± std deviation. (**B**) ^{13}C ILV side chain dynamics ($T_{1\rho}$) comparison between $PBP5_{T485}$:penG (1:8 ratio) and $PBP5_{M485}$:penG (1:8 ratio). Error bars are based on repeat measurements and are often smaller than the symbols used. average ± std deviation. (**C-E**) Changes in $T_{1\rho}$ dynamics mapped on the PBP5 TP domain structure – more residues in $PBP5_{A485}$ have increased dynamics when compared with $PBP5_{M485}$ and $PBP5_{T485}$, when bound to penG.

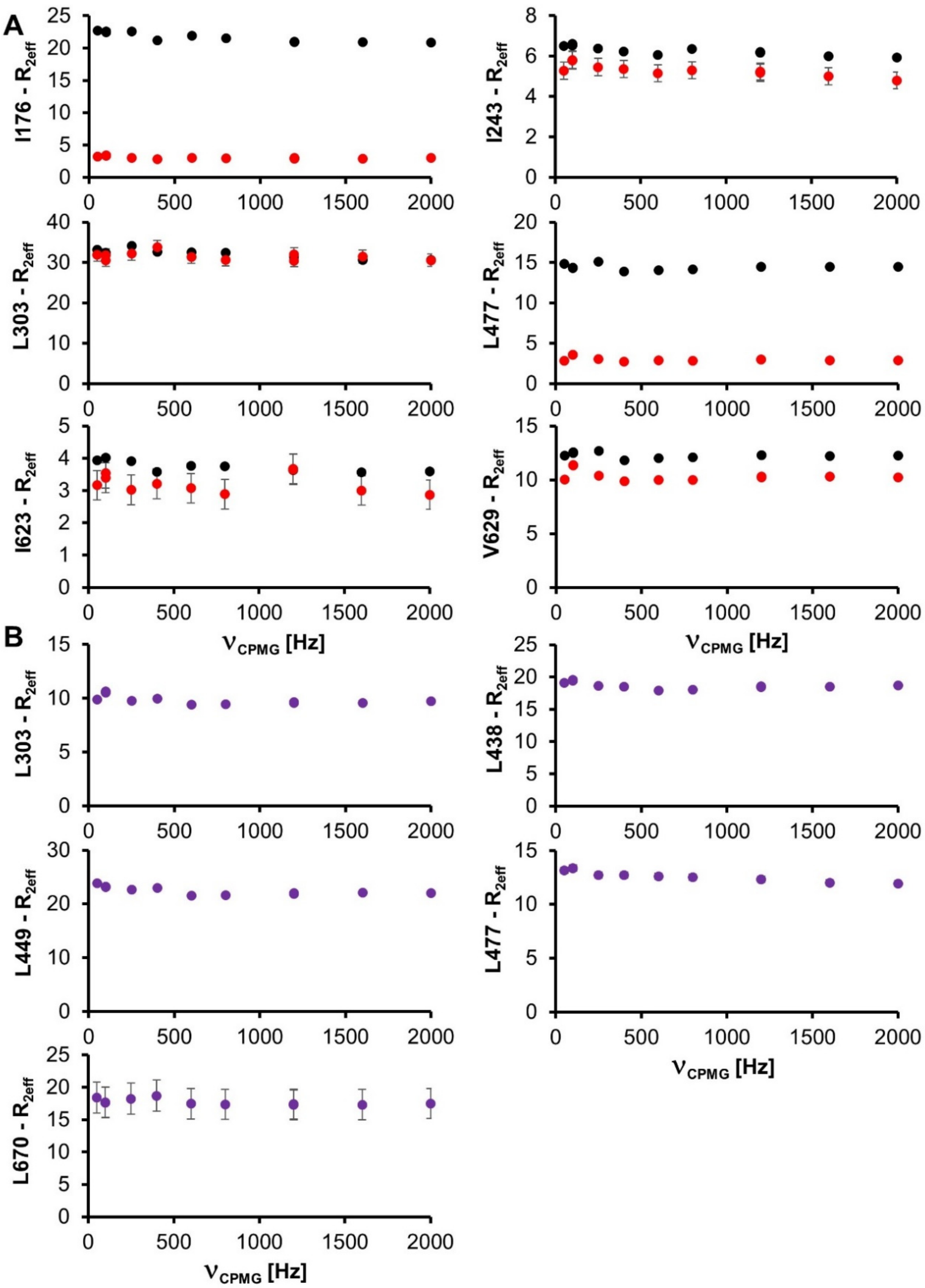

Supplementary Figure 14: ^{13}C ILV ct-Carr-Purcell-Meiboom-Gill (CPMG) data. (**A**) Examples of ^{13}C ILV ct-CPMG relaxation data for residues with increased fast-timescale ^{13}C R1ρ relaxation. Comparison of data for $PBP5_{T485}$:PenG (black) and $PBP5_{A485}$:PenG (red) shows no intermediate timescale motions. (**B**) Examples of ^{13}C ILV ct-CPMG relaxation data for residues with increased fast-timescale ^{13}C R1ρ relaxation. Data for $PBP5_{iS466/A485}$:PenG (violet) shows no intermediate timescale motions. Source data are provided as a Source Data file.

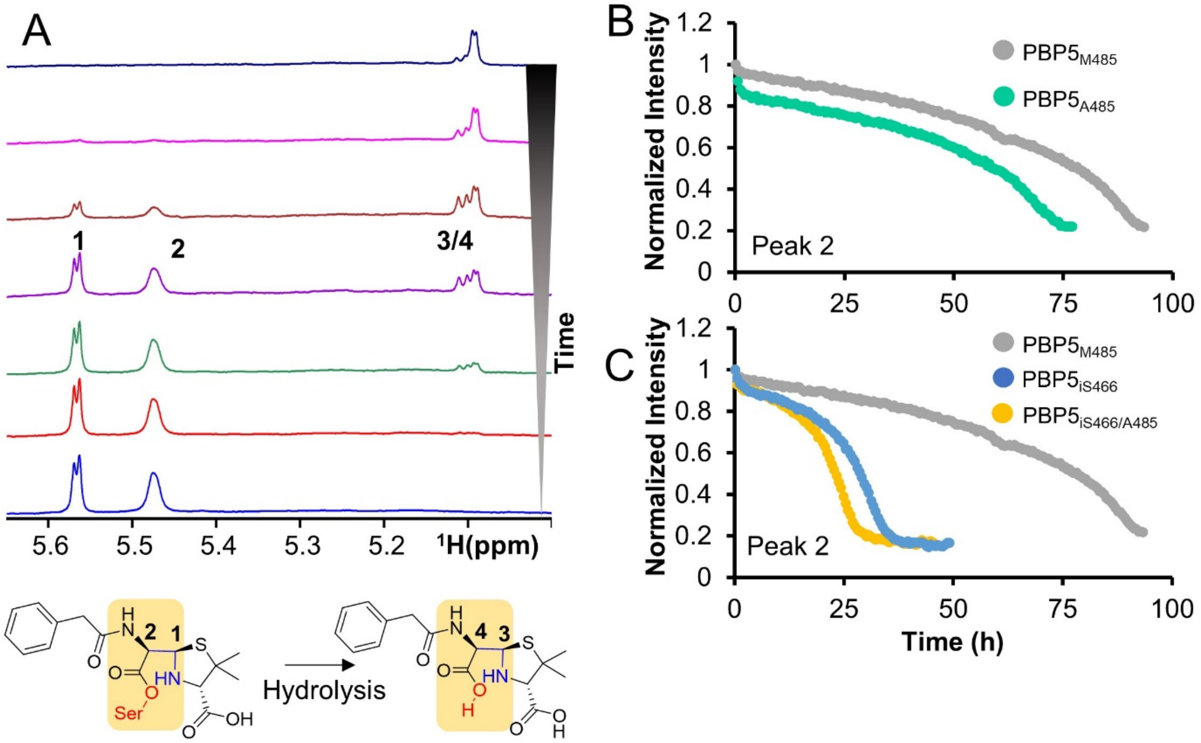

Supplementary Figure 15: Hydrolysis of penicillin. (**A**) PBP5-pencilin-acyl-complex formation can be monitored by 1D ^{1}H NMR spectroscopy. Upon formation, two specific penicillin peaks can be identified in the 1D ^{1}H NMR; Peak 1: 5.59 ppm; Peak 2: 5.49 ppm (annotated in spectrum and on structure – highlighted by yellow box). During hydrolysis Peak 1 and Peak 2 intensities vanish while new peaks appear at 5.1 ppm (assigned as Peaks 3/4). The change in chemical environment of peak1/2 upon hydrolysis leads to an up-field change in the chemical shifts of peaks 3/4 and thus can be followed. (**B**) PBP5-pencilin-acyl-complex hydrolysis followed by the reduction of the Peak 2 intensities, as described in (A) of $PBP5_{M485}$ and $PBP5_{A485}$. (**C**) PBP5-pencilin-acyl-complex hydrolysis followed by the reduction of the Peak 2 intensities, as described in (A) of $PBP5_{M485}$ and $PBP5_{iS466}$ and $PBP5_{iS466/A485}$. Source data are provided as a Source Data file.

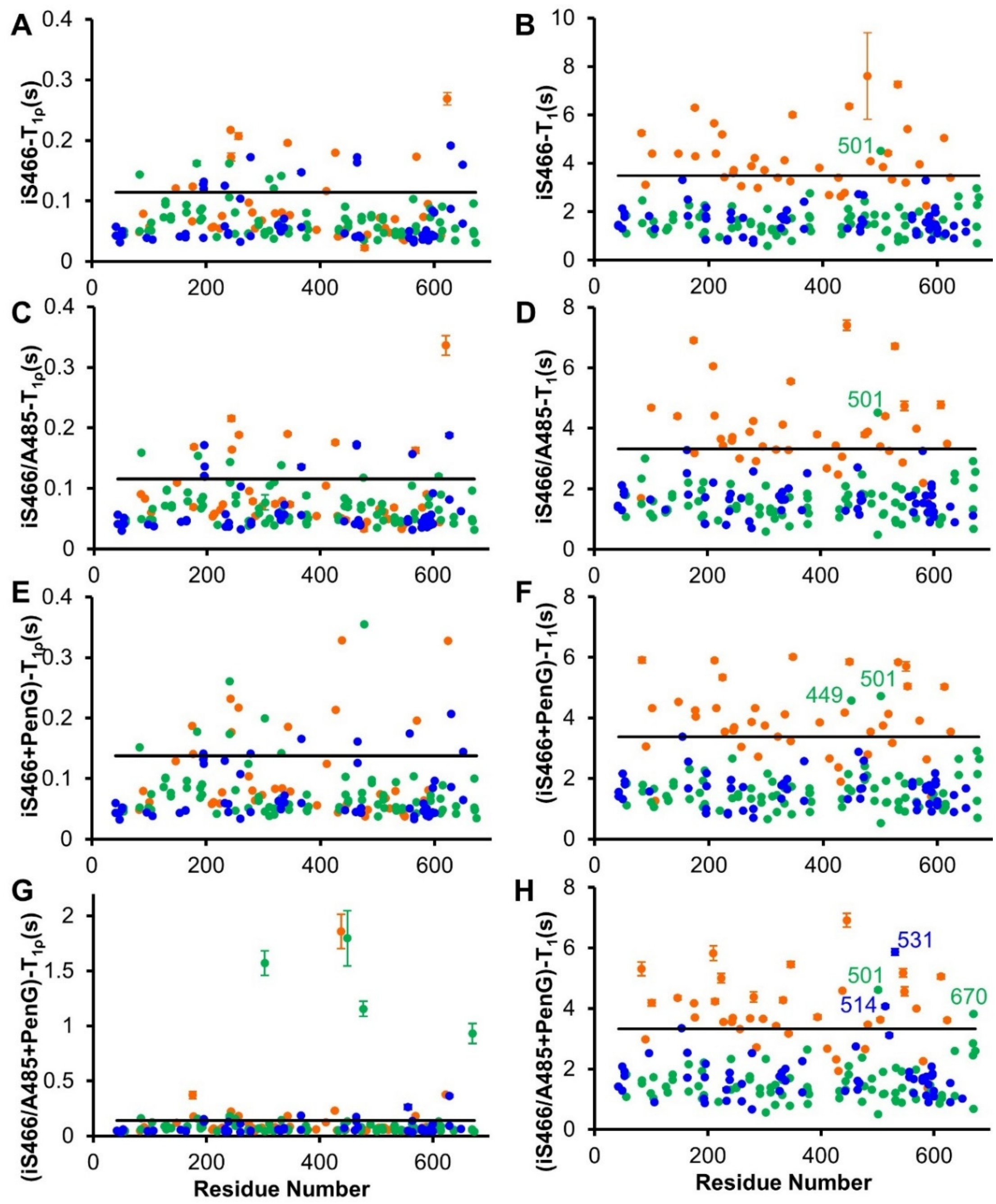

Supplementary Figure 16: ^{13}C ILV T_1 and $T_{1\rho}$ of PBP5 iS466 and iS466/A485 variants in the presence and absence of penG. (**A**) PBP5 iS466 ^{13}C ILV $T_{1\rho}$ vs residue number. (**B**) PBP5 iS466 ^{13}C ILV T_1 vs residue number. (**C**) PBP5 iS466/A485 ^{13}C ILV $T_{1\rho}$ vs residue number. (**D**) PBP5 iS466/A485 ^{13}C ILV T_1 vs residue number. (**E**) PBP5 iS466+saturating penG ^{13}C ILV $T_{1\rho}$ vs residue number. (**F**) PBP5 iS466+saturating penG ^{13}C ILV T_1 vs residue number. (**G**) PBP5 iS466/A485+saturating penG ^{13}C ILV $T_{1\rho}$ vs residue number. (**H**) PBP5 iS466/A485+saturating penG ^{13}C ILV T_1 vs residue number. All graphs: Ile: orange; Val: blue; Leu: green; black line average + standard deviation of T_1. Error bars are based on repeat measurements and are often smaller than the symbols used. average ± std deviation. Source data are provided as a Source Data file.

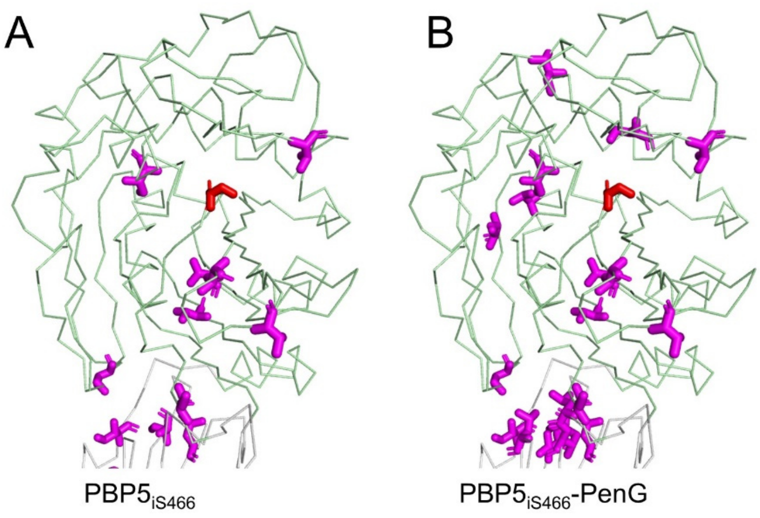

Supplementary Figure 17: PBP5$_{iS466}$ fast timescale ^{13}C ILV dynamics. Changes in ^{13}C ILV $T_{1\rho}$ dynamics mapped on the PBP5 TP domain structure – PBP5$_{iS466}$ (**A**) and PBP5$_{iS466}$:penG (**B**).

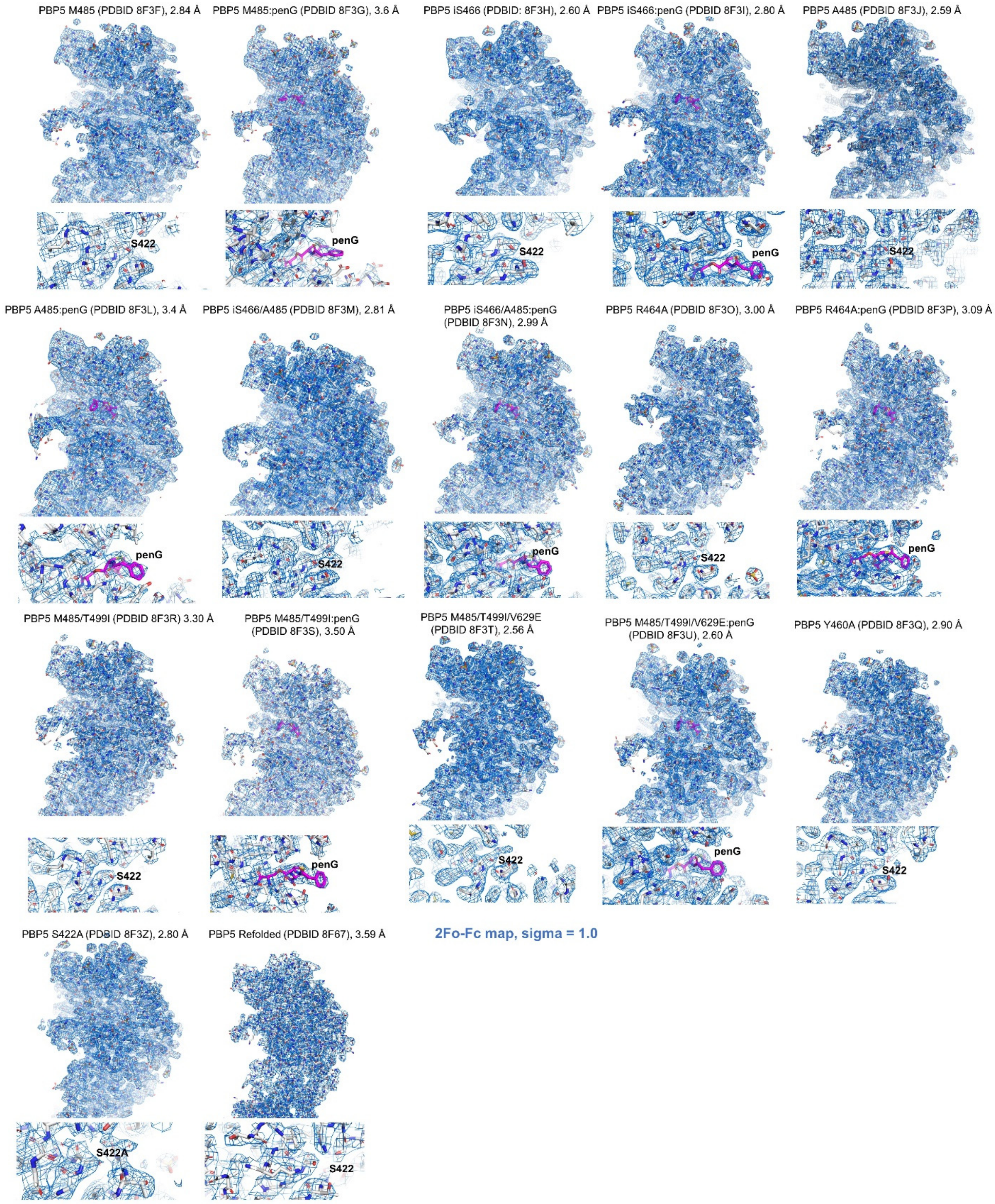

Supplementary Figure 18: 2Fo-Fc maps (sigma = 1) for all crystal structures newly reported in this manuscript (details in Supplementary Table 1).

Supplementary Table 1: Data collection and refinement statistics (molecular replacement) for PBP5 variants with or without penG.

	**PBP5 M485**	**PBP5 M485:penG**	**PBP5 A485**
PDBID	8F3F	8F3G	8F3J
**Data collection**			
Space group	$P6_322$	$P6_322$	$P6_322$
Cell dimensions			
a, *b*, *c* (Å)	192.67, 192.67, 155.86	191.70, 191.70, 156.68	191.13, 191.13, 156.51
α, β, γ (°)	90, 90, 120	90, 90, 120	90, 90, 120
Resolution (Å)	39.79-2.84 (2.96-2.84)	39.70-3.59 (3.93-3.59)	39.60-2.59 (2.67-2.59)
$R_{merge,}$	0.185 (2.006)	0.306 (2.147)	0.212 (2.759)
R_{meas}	0.194 (2.075)	0.314 (2.203)	0.218 (2.878)
$I/\sigma(I)$	12.2 (2.1)	10.1 (2.2)	13.0 (1.5)
$CC_{1/2}$	0.865 (0.744)	0.997 (0.796)	0.997 (0.475)
Completeness (%)	99.4 (95.1)	99.6 (98.7)	99.7 (96.8)
Redundancy	15.1 (14.9)	19.5 (19.5)	19.0 (12.2)
**Refinement**			
Resolution (Å)	39.79-2.84 (2.88-2.84)	38.12-3.59 (3.69-3.59)	38.08-2.59 (2.62-2.59)
No. reflections	39038	20271	52238
R_{work} / R_{free}	0.17(0.37)/0.21(0.40)	0.20(0.34)/0.23(0.42)	0.20 (0.44)/0.22 (0.40)
No. atoms	4978	4650	5037
Protein	4788	4516	4825
Ligand/ion	80	125	80
Water	110	9	132
B factors			
Protein	67.89	123.27	59.89
Ligand/ion	92.44	162.92	74.89
Water	52.82	92.13	49.10
R.M.S. deviations			
Bond lengths (Å)	0.005	0.001	0.002
Bond angles (°)	0.696	0.406	0.504

Values in parentheses are for highest-resolution shell

	PBP5 A485:penG	PBP5 iS466	PBP5 iS466:penG
PDBID	8F3L	8F3H	8F3I
**Data collection**			
Space group	$P6_322$	$P6_322$	$P6_322$
Cell dimensions			
a, *b*, *c* (Å)	193.18, 193.18, 156.40	193.92, 193.92, 154.81	193.62, 193.62, 155.60
α, β, γ (°)	90, 90, 120	90, 90, 120	90, 90, 120
Resolution (Å)	39.10-3.39 (3.63-3.39)	38.70-2.60 (2.68-2.60)	38.90-2.80 (2.90-2.80)
$R_{merge,}$	0.276 (3.385)	0.113 (1.823)	0.098 (1.049)
R_{meas}	0.283 (3.473)	0.122 (1.904)	0.103 (1.099)
$I/\sigma(I)$	10.7 (2.2)	18.9 (1.5)	18.4 (2.7)
$CC_{1/2}$	0.997 (0.662)	0.974 (0.998)	0.999 (0.815)
Completeness (%)	99.7 (98.5)	99.9 (99.1)	99.7 (99.0)
Redundancy	19.6 (20.1)	12.9 (13.0)	11.1 (11.2)
**Refinement**			
Resolution (Å)	39.10-3.40 (3.48-3.40)	38.70-2.60 (2.65-2.60)	38.90-2.80 (2.83-2.80)
No. reflections	24201	49962	42785
R_{work} / R_{free}	0.18 (0.32)/0.20 (0.37)	0.19 (0.36)/0.22 (0.42)	0.18 (0.31)/0.21 (0.35)
No. atoms	4684	5259	5056
Protein	4622	4929	4781
Ligand/ion	40	110	130
Water	22	220	145
B factors			
Protein	127.06	70.63	72.09
Ligand/ion	160.65	101.77	92.05
Water	92.97	58.56	58.16
R.M.S. deviations			
Bond lengths (Å)	0.002	0.002	0.002
Bond angles (°)	0.436	0.479	0.439

Values in parentheses are for highest-resolution shell

	PBP5 iS466/A485	PBP5 iS466/A485:penG	PBP5 R464A
PDBID	8F3M	8F3N	8F3O
**Data collection**			
Space group	$P6_322$	$P6_322$	$P6_322$
Cell dimensions			
a, *b*, *c* (Å)	192.84, 192.84, 156.21	192.26, 192.26, 156.95	192.02, 192.02, 155.38
α, β, γ (°)	90, 90, 120	90, 90, 120	90, 90, 120
Resolution (Å)	38.31-2.80 (2.91-2.80)	39.24-2.99 (3.14-2.99)	39.97-3.00 (3.14-3.00)
$R_{merge,}$	0.138 (0.679)	0.131 (0.542)	0.220 (1.756)
R_{meas}	0.145 (0.754)	0.137 (0.591)	0.226 (1.801)
$I/\sigma(I)$	23.6 (4.5)	13.5 (2.3)	14.1 (2.1)
$CC_{1/2}$	0.925 (0.858)	0.971 (0.964)	0.997 (0.747)
Completeness (%)	95.5 (95.3)	99.6 (99.0)	99.9 (99.7)
Redundancy	23.1 (22.4)	13.1 (13.0)	19.9 (20.3)
**Refinement**			
Resolution (Å)	38.31-2.81 (2.88-2.81)	38.20-2.99 (3.04-2.99)	39.66-3.00 (3.04-3.00)
No. reflections	38448	33794	34317
R_{work} / R_{free}	0.17 (0.25)/0.20 (0.29)	0.20 (0.43)/0.22 (0.49)	0.20 (0.38)/0.23 (0.42)
No. atoms	5214	4832	4940
Protein	4909	4707	4755
Ligand/ion	90	95	90
Water	215	30	95
B factors			
Protein	54.06	79.33	74.18
Ligand/ion	76.55	97.26	88.13
Water	45.79	59.77	50.55
R.M.S. deviations			
Bond lengths (Å)	0.006	0.001	0.001
Bond angles (°)	0.806	0.414	0.420

Values in parentheses are for highest-resolution shell

	PBP5 R464A:penG	PBP5 M485/T499I	PBP5 M485/T499I:penG
PDBID	8F3P	8F3R	8F3S
**Data collection**			
Space group	$P6_322$	$P6_322$	$P6_322$
Cell dimensions			
a, *b*, *c* (Å)	191.74, 191.74, 156.39	191.60, 191.60, 156.32	193.34, 193.34, 156.99
α, β, γ (°)	90, 90, 120	90, 90, 120	90, 90, 120
Resolution (Å)	39.68-3.09 (3.26-3.09)	39.08-3.30 (3.52-3.30)	38.41-3.50 (3.78-3.50)
$R_{merge,}$	0.288 (2.256)	0.165 (0.774)	0.507 (4.077)
R_{meas}	0.295 (2.314)	0.170 (0.794)	0.521 (4.184)
$I/\sigma(I)$	13.2 (2.3)	20.3 (7.2)	11.2 (3.9)
$CC_{1/2}$	0.996 (0.865)	0.995 (0.949)	0.992 (0.846)
Completeness (%)	99.7 (98.5)	99.8 (99.6)	99.3 (99.7)
Redundancy	19.9 (20.2)	19.6 (19.9)	19.6 (19.9)
**Refinement**			
Resolution (Å)	38.10-3.09 (3.15-3.09)	36.99-3.30 (3.37-3.30)	37.31-3.50 (3.59-3.50)
No. reflections	31305	26017	22060
R_{work} / R_{free}	0.19 (0.34)/0.21 (0.38)	0.18 (0.29)/0.20 (0.31)	0.18 (0.30)/0.21 (0.32)
No. atoms	4918	4828	4811
Protein	4739	4724	4720
Ligand/ion	80	75	75
Water	99	29	16
B factors			
Protein	72.56	68.08	104.97
Ligand/ion	97.14	83.42	129.85
Water	57.60	44.47	62.66
R.M.S. deviations			
Bond lengths (Å)	0.002	0.001	0.001
Bond angles (°)	0.492	0.388	0.402

Values in parentheses are for highest-resolution shell

	PBP5 M485/T499I/V629E	PBP5 M485/T499I/V629E: penG	PBP5 Y460A
PDBID	8F3T	8F3U	8F3Q
**Data collection**			
Space group	$P6_322$	$P6_322$	$P6_322$
Cell dimensions			
a, *b*, *c* (Å)	192.78, 192.78, 155.43	194.30, 194.30, 154.41	192.44, 192.44, 156.27
α, β, γ (°)	90, 90, 120	90, 90, 120	90, 90, 120
Resolution (Å)	39.78-2.56 (2.63-2.56)	38.60-2.60 (2.68-2.60)	39.78-2.89 (3.02-2.89)
$R_{merge,}$	0.068 (1.139)	0.077 (1.102)	0.184 (1.926)
R_{meas}	0.072 (1.193)	0.080 (1.153)	0.189 (1.976)
$I/\sigma(I)$	23.8 (2.2)	20.2 (2.3)	15.9 (1.9)
$CC_{1/2}$	0.999 (0.739)	0.999 (0.801)	0.998 (0.736)
Completeness (%)	99.9 (99.0)	99.9 (98.8)	99.7 (98.8)
Redundancy	11.2 (11.2)	11.5 (11.4)	19.9 (20.0)
**Refinement**			
Resolution (Å)	39.78-2.56 (2.59-2.56)	38.60-2.60 (2.63-2.60)	39.78-2.90 (2.94-2.90)
No. reflections	55220	53219	38192
R_{work} / R_{free}	0.19 (0.33)/0.21 (0.36)	0.20 (0.35)/0.22 (0.42)	0.20 (0.38)/0.22 (0.36)
No. atoms	5223	5202	4996
Protein	4869	4863	4756
Ligand/ion	98	105	110
Water	256	234	130
B factors			
Protein	74.12	80.18	73.39
Ligand/ion	88.39	99.43	88.90
Water	67.57	66.87	58.30
R.M.S. deviations			
Bond lengths (Å)	0.001	0.001	0.001
Bond angles (°)	0.412	0.434	0.420

Values in parentheses are for highest-resolution shell.

	PBP5 S422A	PBP5 T485 refolded
PDBID	8F3Z	8F67
**Data Collection**		
Space group	$P6_322$	$C222_1$
Cell dimensions		
a, *b*, *c* (Å)	190.69, 190.69, 156.11	190.94, 331.67, 156.18
α, β, γ (°)	90, 90, 120	90, 90, 90
Resolution (Å)	39.50-2.80 (2.91-2.80)	39.63-3.59 (3.68-3.59)
$R_{merge,}$	0.351 (3.355)	0.293 (1.096)
R_{meas}	0.360 (3.447)	0.317 (1.197)
$I/\sigma(I)$	14.3 (3.3)	8.0 (3.5)
$CC_{1/2}$	0.995 (0.616)	0.977 (0.605)
Completeness (%)	99.8 (99.2)	99.3 (92.8)
Redundancy	19.8 (19.2)	6.8 (6.3)
**Refinement**		
Resolution (Å)	39.03-2.80 (2.83-2.80)	39.63-3.59 (3.65-3.59)
No. reflections	41592	57654
R_{work} / R_{free}	0.18 (0.31)/0.21 (0.35)	0.18 (0.23)/0.21 (0.26)
No. atoms	5124	14680
Protein	4772	14222
Ligand/ion	120	330
Water	232	128
B factors		
Protein	52.84	69.24
Ligand/ion	76.52	100.19
Water	40.91	39.96
R.M.S. deviations		
Bond lengths (Å)	0.002	0.002
Bond angles (°)	0.478	0.430

Values in parentheses are for highest-resolution shell.

Supplementary Table 2: Summary of all PBP5 variants produced for ILV assignment. Folded/misfolded assignment was given based on the 2D [^{1}H,^{13}C] HMQC ILV spectrum of the variant when compared to the 2D [^{1}H,^{13}C] HMQC ILV spectrum of PBP5$_{T485}$.

**Residue**	*Notes*	**Residue**	*Notes*	**Residue**	*Notes*
V49A	*misfolded*	L332I	folded	L543A	folded
V49I	folded	I333A	folded	I545A	folded
L56A	*misfolded*	I343A	folded	I548A	folded
L56I	folded	L345A	folded	V556A	folded
I83V	folded	I347A	folded	L563A	folded
L99A	*misfolded*	L377A	folded	V564A	folded
L99I	folded	I427A	folded	I569A	folded
I147A	*misfolded*	I431A	folded	V580A	folded
L153A	*misfolded*	L433A	folded	I581A	folded
L153I	folded	I438A	folded	V586I	folded
I176A	*misfolded*	I446A	folded	I589A	folded
I176V	folded	L449A	folded	*V590A*	*misfolded*
L184A	*misfolded*	V462A	folded	V590L	folded
L184I	folded	V465A	folded	V596A	folded
V195A	folded	V468A	folded	V600A	folded
I213A	folded	V471A	folded	L607A	folded
V233A	folded	L477A	folded	I612A	folded
V239A	folded	I478A	folded	L614A	folded
I243A	folded	I483A	folded	I623A	folded
L274A	folded	I505A	folded	V629E	folded
I275A	folded	L510A	folded	L637A	folded
V278A	folded	I514A	folded	L669A	folded
I281A	folded	I521A	folded	L670A	folded
L303A	folded	I531A	folded		
L332A	*misfolded*	L532A	folded		

Supplementary Table 3: Average NMR relaxation rates of PBP5 variants; Ile: orange; Val: blue; Leu: green; Source data are provided as a Source Data file.

		M485	T485	iS466	A485	iS466/A485
T_1	All	2.16±1.32	2.11±1.3	2.16±1.33	2.36±1.49	2.08±1.23
	Ile	4.05±1.37	4.48±1.31	4.14±1.29	4.53±1.63	3.88±1.26
	Leu	1.56±0.62	1.53±0.61	1.58±0.61	1.715±0.68	1.54±0.61
	Val	1.65±0.53	1.60±0.51	1.61±0.56	1.823±0.64	1.63±0.55
$T_{1\rho}$	All	0.061±0.04	0.067±0.04	0.072±0.04	0.105±0.08	0.073±0.04
	Ile	0.082±0.06	0.088±0.06	0.044±0.06	0.134±0.11	0.093±0.06
	Leu	0.058±0.03	0.063±0.03	0.021±0.03	0.102±0.06	0.069±0.03
	Val	0.053±0.03	0.058±0.03	0.033±0.04	0.089±0.07	0.065±0.04
T_2	All	0.052±0.03	0.056±0.03	0.061±0.04	0.089±0.07	0.062±0.04
	Ile	0.065±0.04	0.07±0.04	0.032±0.04	0.107±0.08	0.074±0.05
	Leu	0.046±0.02	0.051±0.02	0.019±0.3	0.082±0.05	0.055±0.03
	Val	0.050±0.03	0.055±0.03	0.032±0.04	0.085±0.07	0.062±0.04

Supplementary Table 4: Average NMR relaxation rates of PBP5 variants in the presence of penG; Ile: orange; Val: blue; Leu: green; Source data are provided as a Source Data file.

with penG		M485	T485	iS466	A485	iS466/A485
T_1	All	2.14±1.28	2.1±1.25	2.12±1.25	2.46±1.57	2.09±1.2
	Ile	3.94±1.28	3.93±1.24	3.89±1.19	4.66±1.78	3.85±1.11
	Leu	1.59±0.69	1.55±0.62	1.59±0.69	1.81±0.76	1.52±0.66
	Val	1.64±0.53	1.64±0.52	1.62±0.54	1.84±0.55	1.76±0.84
$T_{1\rho}$	All	0.064±0.04	0.065±0.04	0.082±0.06	0.097±0.07	0.124±0.24
	Ile	0.089±0.07	0.082±0.05	0.044±0.06	0.134±0.10	0.152±0.28
	Leu	0.059±0.03	0.062±0.03	0.021±0.03	0.09±0.05	0.139±0.29
	Val	0.054±0.03	0.058±0.03	0.033±0.04	0.083±0.06	0.081±0.06
T_2	All	0.054±0.04	0.055±0.03	0.069±0.05	0.082±0.06	0.072±0.05
	Ile	0.072±0.05	0.066±0.04	0.085±0.06	0.108±0.07	0.126±0.25
	Leu	0.047±0.03	0.050±0.02	0.061±0.05	0.073±0.05	0.122±0.28
	Val	0.051±0.03	0.055±0.03	0.069±0.04	0.079±0.06	0.076±0.06
