# Supplementary material for: Molecular basis of β-lactam antibiotic resistance of ESKAPE bacterium E. faecium Penicillin Binding Protein PBP5
Source: Nat Commun. 2023 Jul 17;14:4268. doi: 10.1038/s41467-023-39966-5 (PMC10352307; doi:10.1038/s41467-023-39966-5)
Supplement: Supplementary file 3 — Reporting Summary [file 41467_2023_39966_MOESM3_ESM.pdf]

nature portfolio

Corresponding author(s): Wolfgang Peti

Last updated by author(s): Feb 22, 2023

Reporting Summary

Nature Portfolio wishes to improve the reproducibility of the work that we publish. This form provides structure for consistency and transparency in reporting. For further information on Nature Portfolio policies, see our Editorial Policies and the Editorial Policy Checklist.

Statistics

For all statistical analyses, confirm that the following items are present in the figure legend, table legend, main text, or Methods section.

n/a	Confirmed	
☐	☒	The exact sample size (*n*) for each experimental group/condition, given as a discrete number and unit of measurement
☐	☒	A statement on whether measurements were taken from distinct samples or whether the same sample was measured repeatedly
☐	☒	The statistical test(s) used AND whether they are one- or two-sided<br>*Only common tests should be described solely by name; describe more complex techniques in the Methods section.*
☒	☐	A description of all covariates tested
☐	☒	A description of any assumptions or corrections, such as tests of normality and adjustment for multiple comparisons
☐	☒	A full description of the statistical parameters including central tendency (e.g. means) or other basic estimates (e.g. regression coefficient) AND variation (e.g. standard deviation) or associated estimates of uncertainty (e.g. confidence intervals)
☒	☐	For null hypothesis testing, the test statistic (e.g. *F*, *t*, *r*) with confidence intervals, effect sizes, degrees of freedom and *P* value noted<br>*Give P values as exact values whenever suitable.*
☒	☐	For Bayesian analysis, information on the choice of priors and Markov chain Monte Carlo settings
☒	☐	For hierarchical and complex designs, identification of the appropriate level for tests and full reporting of outcomes
☒	☐	Estimates of effect sizes (e.g. Cohen's *d*, Pearson's *r*), indicating how they were calculated

Our web collection on statistics for biologists contains articles on many of the points above.

Software and code

Policy information about availability of computer code

Data collection	Topspin 4.1, AutoXDS (XDS, VERSION Nov 11, 2017 BUILT=20180124), Clariostar SMART Control 6.2
Data analysis	GraphPad Prism 9.5, CARA 1.8.4.2, CcPNMR 2.5.2, NMRFARM SPARKY 1.47, SigmaPlot 14.5, Microsoft® Excel® for Microsoft 365 MSO (Version 2301 Build 16.0.16026.20002) 64-bit, Phenix 1.19.1-4122, Coot 0.92, Clariostar Mars 3.42, PyMol 2.4.1, NMRviewJ 9.2,

For manuscripts utilizing custom algorithms or software that are central to the research but not yet described in published literature, software must be made available to editors and reviewers. We strongly encourage code deposition in a community repository (e.g. GitHub). See the Nature Portfolio guidelines for submitting code & software for further information.

Data

Policy information about availability of data

All manuscripts must include a data availability statement. This statement should provide the following information, where applicable:

- Accession codes, unique identifiers, or web links for publicly available datasets
- A description of any restrictions on data availability
- For clinical datasets or third party data, please ensure that the statement adheres to our policy

All NMR chemical shifts have been deposited in the BioMagResBank (BMRB 51690 [; https://bmrb.io/data_library/summary/?bmrbId=51690https://bmrb.io/data_library/summary/?bmrbId=51690] (Sequence Specific 1H, 13C, and 15N backbone resonance assignments of 70 kDa Penicillin Binding Protein PBP5) and BMRB; 51692; [https://bmrb.io/data_library/summary/?bmrbId=51692https://bmrb.io/data_library/summary/?bmrbId=51692] (Sidechain Ile, Leu, and Val methyl

chemical shift assignments for Penicillin Binding Protein 5)). Atomic coordinates and structure factors have been deposited in the Protein Data Bank (PDB: 8F3F [https://doi.org/10.2210/pdb8F3F/pdb], 8F3G [https://doi.org/10.2210/pdb8F3G/pdb], 8F3H [https://doi.org/10.2210/pdb8F3H/pdb], 8F3I [https://doi.org/10.2210/pdb8F3I/pdb], 8F3J [https://doi.org/10.2210/pdb8F3J/pdb], 8F3L [https://doi.org/10.2210/pdb8F3L/pdb], 8F3M [https://doi.org/10.2210/pdb8F3M/pdb], 8F3N [https://doi.org/10.2210/pdb8F3N/pdb], 8F3O [https://doi.org/10.2210/pdb8F3O/pdb], 8F3P [https://doi.org/10.2210/pdb8F3P/pdb], 8F3Q [https://doi.org/10.2210/pdb8F3Q/pdb], 8F3R [https://doi.org/10.2210/pdb8F3R/pdb], 8F3S [https://doi.org/10.2210/pdb8F3S/pdb], 8F3T [https://doi.org/10.2210/pdb8F3T/pdb], 8F3U [https://doi.org/10.2210/pdb8F3U/pdb], 8F3Z [https://doi.org/10.2210/pdb8F3Z/pdb], 8F67 [https://doi.org/10.2210/pdb8F67/pdb], 6MKA [https://doi.org/10.2210/pdb6MKA/pdb], 6MKG [https://doi.org/10.2210/pdb6MKG/pdb]).). All other data supporting the findings ofgenerated this study are provided as source data with this paper and are provided atalso available at Figshare (10.6084/m9.figshare.22773818).

Human research participants

Policy information about studies involving human research participants and Sex and Gender in Research.

Reporting on sex and gender	N/A
Population characteristics	N/A
Recruitment	N/A
Ethics oversight	N/A

Note that full information on the approval of the study protocol must also be provided in the manuscript.

Field-specific reporting

Please select the one below that is the best fit for your research. If you are not sure, read the appropriate sections before making your selection.

☒ Life sciences ☐ Behavioural & social sciences ☐ Ecological, evolutionary & environmental sciences

For a reference copy of the document with all sections, see nature.com/documents/nr-reporting-summary-flat.pdf

Life sciences study design

All studies must disclose on these points even when the disclosure is negative.

Sample size	SDS-PAGE and fluorescence based assays were done with an n=3 to 6; mean +- STD was used for statistical analysis as commonly used for these techniques. No statistical method was used to determine sample sizes and no sample size calculation was performed. The standard in the research field is to sample n >=3 independent measurements. Consequently, at least three independent replicates were performed for each condition in all biochemical experiments and biophysics measurements. NMR measurements were averaged over at least 4 independent transients (scans). When multiple PBP5 structures were determined at a variety of resolution - highest resolution structure is reported (all structures were identical).
Data exclusions	No data was excluded in our analysis.
Replication	All data was recorded multiple times to reproduce the data (repeats are indicated throughout the manuscript/figure captions and methods). Data was also collected from different protein expression/purification batches, i.e., not just technical replicates, but also experimental replicates. All attempts of data collection were successful. All NMR experiments were at least averaged over 4 independent transients (scans).
Randomization	No randomization was necessary for our biochemical studies as sample bias is impossible due to the use of experimental replicates. Experimental parameters were precisely controlled and all ingredients were standardized.
Blinding	Blinding was not relevant for the study as no test subjects were used in this study as typical done for all published biochemical studies. Again experimental conditions where precisely controlled.

Reporting for specific materials, systems and methods

We require information from authors about some types of materials, experimental systems and methods used in many studies. Here, indicate whether each material, system or method listed is relevant to your study. If you are not sure if a list item applies to your research, read the appropriate section before selecting a response.

Materials & experimental systems

n/a	Involved in the study
☒	☐ Antibodies
☒	☐ Eukaryotic cell lines
☒	☐ Palaeontology and archaeology
☒	☐ Animals and other organisms
☒	☐ Clinical data
☒	☐ Dual use research of concern

Methods

n/a	Involved in the study
☒	☐ ChIP-seq
☒	☐ Flow cytometry
☒	☐ MRI-based neuroimaging
